# Editorial: Viral Infection at the Maternal-Fetal Interface

**DOI:** 10.3389/fimmu.2022.828681

**Published:** 2022-02-04

**Authors:** Abhay P. S. Rathore, Vivian Vasconcelos Costa, Ashley L. St. John

**Affiliations:** ^1^ Department of Pathology, Duke University Medical Center, Durham, NC, United States; ^2^ Research Group in Arboviral Diseases, Institute of Biological Sciences, Universidade Federal de Minas Gerais, Belo Horizonte, Brazil; ^3^ Center for Drug Research and Development of Pharmaceuticals, Institute of Biological Sciences, Universidade Federal de Minas Gerais, Belo Horizonte, Brazil; ^4^ Departament of Morphology, Institute of Biological Sciences, Universidade Federal de Minas Gerais, Belo Horizonte, Brazil; ^5^ Program in Emerging Infectious Diseases, Duke-National University of Singapore (NUS), Singapore, Singapore; ^6^ Department of Microbiology and Immunology, Yong Loo Lin School of Medicine, National University of Singapore (NUS), Singapore, Singapore; ^7^ SingHealth Duke-National University of Singapore (NUS) Global Health Institute, Singapore, Singapore

**Keywords:** viruses, maternal-fetal, COVID - 19, Zika, CMV, HIV, maternal immunity

The emergence of infections such as Zika virus (ZIKV) has highlighted the importance of understanding diseases that can be transferred vertically from mother to the fetus. Viruses constitute a large proportion of TORCH pathogens [Toxoplasmosis, Other (syphilis, varicella-zoster, parvovirus B19, Zika), Rubella, Cytomegalovirus (CMV), and Herpes infections], which can result in congenital infections impacting neonatal health and neurodevelopment. Maternal and fetal immunity are critical for infection clearance, while pathogens have evolved to evade both innate and adaptive immune responses that restrict infection spread. In this collection of research articles and reviews, researchers have discussed the interplay between a mothers’ immunity and the fetal developing immune system as well as the consequences to fetal health during congenital viral infections.

The human full-term pregnancy is approximately 40 weeks and divided in to three trimesters reflecting stages of development. Gestational age at the time of exposure to infections is one of the important determinants of congenital disease outcomes. For example, ZIKV infection during the first trimester of pregnancy is associated with a higher risk of developing birth defects such as microcephaly and ocular defects in newborns (1). This was elegantly supported by Newman et al. in a study mimicking the sexual transmission of ZIKV infection by intravaginal inoculation of ZIKV in rhesus macaques during developmental time points consistent with the first trimester of pregnancy. The pregnant females exposed to ZIKV during early gestation were found to have non-viable embryos and viral RNA was detected in the demised embryos, supporting a risk of pregnancy loss during early gestation. In contrast to ZIKV, CMV infections are associated with adverse fetal health outcomes when infection occurs during the late gestation periods of second-third trimester (2). To address the increased susceptibility of CMV infection during late gestation, Berkebile et al. used a Guinea pig model of CMV infection. When guinea pigs were infected at days 21 and 35 of gestation (equivalent to mid to late gestational time points in humans), maternal and placental viral load did not differ between the two infection groups. However, transcriptome analysis revealed significant changes in transcripts associated with immune activation in the gestational day 35 infection group suggesting placental sensitization to injury during late gestation CMV infection. In contrast to ZIKV infection, which is acute and is mainly transferred *in utero* (3), HIV transmission from mother to child can occur during various stages of pregnancy and at the time of birth or after birth. This complex nature of HIV transmission from mother to child was reviewed for this collection by Amin et al. They elaborately discussed HIV infection in the context of early life immune responses and viral persistence. Together these studies emphasize the importance of infection timing during pregnancy and early life to immune and functional outcomes of viral infections.

The placenta is a unique organ that emerges in the context of pregnancy to provide nourishment and gas exchange for the developing fetus (4). This critical role is balanced by the need for immune functions that protect mother and developing fetus from adverse outcomes, including congenital infections. The structure of the placenta is central to this role, with the maternal decidua segregated from the placenta through a network of tight barriers involving the syncytiotrophoblast cells and fetal endothelium, where active transport processes are involved in allowing specific substances and molecules to pass from mother to fetus. The complexities of the maternal-fetal interactions at the placental interface are emphasized by the presence of the fully functional maternal immune system, which must interact with newly developing fetal immunity. Thus, during healthy pregnancies, immune responses at the maternal-fetal interface are characterized by immune tolerance (5), which must be balanced with the need to provide protection from pathogens. This complexity in the maternal immune system is highlighted by Moström et al. utilizing a pregnancy model of rhesus macaques. Various innate and adaptive cell populations were profiled in the maternal decidua and in the peripheral blood of pregnant mothers. When comparing healthy and ZIKV-infected pregnancies, signatures of immune suppression with reduced recruitment of functional cytotoxic T cells were observed in ZIKV-infected pregnancies, suggesting reduced capacity for infection clearance.

Viral infection at the maternal-fetal interface could cause placental insufficiency resulting in intra uterine growth restriction (IUGR) and thereby limiting normal fetal growth (6). Experimentally, using wildtype and type-I interferon deficient mice, Andrade et al. show that ZIKV infection resulted in the release of proinflammatory mediators and disrupted the abca1 transporter in the placenta, leading to placental insufficiency and IUGR. In humans, Ronchi et al. utilized morphometric analysis of placenta tissue from ZIKV- and HIV-infected pregnant women. Their data revealed pathological similarities such as increased numbers of knots, sprouts, and CD163^+^ Hofbauer cell hyperplasia with more pronounced Hofbauer cell hyperplasia in ZIKV compared to HIV infected pregnancies. Morphologic features of the placenta were also investigated by Rebutini et al. in the context of maternal SARS-CoV-2 infection. Although SARS-CoV-2 is not well established to vertically transmit from mother to fetus (7, 8), this report highlights its potential of maternal viral infection to influence fetal outcomes by impacting the placental tissue. Strikingly, the authors observed both maternal and fetal malperfusion during COVID-19, and found that pregnant women with SARS-CoV-2 infection were more likely to display chronic histiocytic intervillositis, which is an inflammatory disorder involving infiltration of histiocytes/macrophages into the placenta (9). These reports providing comparisons of the virus-induced changes to the placental ultrastructure in the context of chronic versus acute viral infections highlight that there are likely similar pathways that are triggered to restrict viral infection during pregnancy at the maternal-fetal interface and that these could influence the health of the placental tissue.

Recognizing breast feeding as another important component of maternal-infant exchange with the potential to influence immunity and infection, García et al. characterized the influence of SARS-CoV-2 infection on immune compounds in breast milk. Using a case control study design, they revealed a profile of immune activation that could be detected in the form of higher levels of pro-inflammatory cytokines such as Eotaxin, IP-10, MIP-1α, and RANTES, as well as several growth factors. The pro-inflammatory profile of breast milk from SARS-CoV-2 convalescent mothers is likely supportive of the role of breast milk in providing immune protection to infants from viral infections to which they are likely to be exposed post-partum.

Maternal adaptive immune responses that are pre-existing also have the potential to influence fetal outcomes during pregnancy. In the context of flavivirus infections, exposure to multiple flaviviruses in a lifetime is common, and these heterologous immune responses are often cross-reactive but non-neutralizing and cannot induce full protection against closely related viruses (10). Antibodies against dengue virus for example, have been shown to enhance replication of ZIKV through both antibody-dependent enhancement of infection (ADE) involving uptake of virus/antibody immune complexes *via* Fc receptors on permissive cells such as monocytes, or through transplacental trafficking using the FcRN receptor (11, 12). Acklin et al. addressed the question of whether West Nile Virus (WNV) antibodies could influence ZIKV pathogenesis in a murine model using STAT2^-/-^ mice to enhance disease severity. They found similar resorption rates, fetal and placental sizes and virus infection levels in the presence of human WNV-specific antibodies in mice, in spite of those antibodies promoting efficient ADE *in vitro*, suggesting WNV-specific antibodies to have negligible influence on enhancement of ZCS.

Adaptive immune responses in donors with a history of ZIKV infection during pregnancy were examined in depth by Badolato-Corrêa et al. Extensive characterization of antigen-specific CD4 and CD8 T cells highlighted the rapid decay of ZIKV-specific CD8 T cells relative to CD4 T cells in the months following infection resolution. Interestingly, the authors highlight that there were not significant differences between the T cell responses of mothers with asymptomatic children, versus children with ZCS. This study suggests that similar memory T cell responses are induced by mild and severe congenital disease in the context of ZIKV infection.

Finally, one study investigated whether interventions could be used to prevent fetal complications or severe outcomes of infection. Marim et al. demonstrated that targeting of fetal kynurenine pathway signaling during ZIKV infection in mice could improve neurological outcomes of congenital infection.

Together the articles in this Research Topic advance our understanding of immunological mechanisms promoting health or disease at the maternal-fetal interface during viral infections.

## Author Contributions

All authors contributed to the article and approved the submitted version.

## Funding

The authors acknowledge funding from NRF-CRP17-2017-04.

## Conflict of Interest

The authors declare that the research was conducted in the absence of any commercial or financial relationships that could be construed as a potential conflict of interest.

## Publisher’s Note

All claims expressed in this article are solely those of the authors and do not necessarily represent those of their affiliated organizations, or those of the publisher, the editors and the reviewers. Any product that may be evaluated in this article, or claim that may be made by its manufacturer, is not guaranteed or endorsed by the publisher.
